# Botanical Description of British Plants

**Published:** 1807-04

**Authors:** 


### *Botanical Description of British Plants.*

[ Continued from page 68—.74 ]

13. Anemone. *A. pulsatilla.*

*Ang.* Pasque flower. Passe flower.

*Gen. Desc.* *As above.*

*Spec. Desc.* *Fruit-st.* with an involucrum. *Seeds* with tails. *Stem* downy, one-flowered. *Flower* at first, covered with inv. then nodding, on a long fruit-stalk. *Invol.* many-cleft, downy. *Petals* downy, smooth within, purple. *Seeds* crowned with hairy styles. *High pastures.* Bl. Apr. May.

*Use.* This plant differs but little from the preceding species, and might probably be a good substitute for it.—*Woodville.* The whole plant is acrid and blisters the skin. The juice of the petals stains paper green. Sheep and goats eat it; horses, cows, and swine refuse it.—*Withering.*

14. Anemone. *A. Nemorosa.*

*Ang.* Wood anemony.

*Gen. Desc.* *As above.*

*Spec. Desc.* *Flowers* naked. *Seeds* tail-less, pointed. *Stem.* smooth 1-fl. purplish. *Stem leaves* doubly three-fold. *Leafits* egg-spear-shaped, snipt, slightly hairy. *Petals* three inner, and three outer, the latter tinged with deep purple underneath. *Woods, hedges, hollow ways.* Bl. April.

*Use.*

*Use.* The whole plant is acrid. When it is eaten by sheep that are unaccustomed to it, it brings on a bloody flux. The flowers fold up in a curious manner against rain.—Goats and sheep eat it; horses, cows, and swine refuse it.—*Linn.* The paper, in which the dried specimens are preserved, is stained brown, whence it appears probable that it might be employed as a dye.—*Stokes.*

15. Thalictrum. *T. flavum.*

*Ang.* Meadow rue-weed.

*Gen. Desc.* Cal. 0. Petals four or five. Capsules many, rather beaked.

*Spec. Desc.* *Stem* furrowed, leafy. *Leafits* acute three-cleft. *Panicle* much branched, upright, compact. *Flowers* upright, yellow white. *Pet.* four. *Stam.* twenty-four. *Pist.* ten to sixteen. *Moist ground.* Bl. June.

*Use.* A cataplasm, made of the leaves, has been known to give relief in the *sciatica.* The root dyes wool yellow. Horses, cows, sheep and goats eat it; swine are not fond of it.—*Linnæus.*

16. Ranunculus. *R. ficaria. Ficaria verna.*

*Ang.* Common pilewort. Lesser celandine.

*Gen. Desc.* Cal. decid. five or three-leaves. Petals five (rarely two, three or eight) with a nectariferous scale, or pore within the claw. Styles permanent. Seeds incrusted, upright.

*Spec. Desc.* *Leaves,* heart shaped, angular, smooth, shining, on leaf-st. *Stem.* 1-fl. *Petals* eight or nine, bright yellow. *Cal.* three-leav. *Root* oblong egg-shaped bulbs. *Pastures.* Bl. Apr.

*Use.* The young leaves may be eaten in the Spring along with other pot-herbs. Goats and sheep eat it; cows and horses refuse it.—*Withering.*

17. Ranunculus. *R. flammula.*

*Ang.* Lesser spearwort.

*Gen. Desc.* *As above.*

*Spec. Desc.* *Leaves* egg-shaped, on leafstalks, toothed or serrated. *Leaf-st.* long, rather a doubling of the leaves. *Flowers* yellow. *Bogs, boggy meadows.* Bl. June, Sept.

*Use.* It is very acrid; and its acrimony rises in distillation. The distilled water operates like white vitriol, as an *instantaneous emetic;* and Dr. Withering asserts from his own experience, that in cases of poison, or other circumstances, where it is desirable to make the patient vomit instantly, it is preferable to any other medicine yet known; and does not excite those painful contractions in the the upper part of the stomach, which are sometimes occasioned by white vitriol, thereby defeating the intention for which it was given. Applied externally, it inflames and blisters the skin. Horses eat it; cows, sheep, goats and swine refuse it.—*Withering.*

18. Ranunculus. *R. Sceleratus.*

*Ang.* Round-leaved water crowfoot. Celery-leaved crowfoot.

*Gen. Desc. As above.*

*Spec. Desc. Lower leaves* hand-shaped; *upper-l.* fingered; *root-l.* kidney-shaped, lobed, on long leaf-stalks, smooth. *Plant* dark green, branched, succulent. *Petals* small, yellow. *Recept.* egg-shaped. *Fruit* oblong. *Shallow waters.* Bl. May, June.

*Use.* The whole plant is very corrosive; and is said to be made use of by beggars, for the purpose of ulcerating their feet, which they thus expose to the public to excite compassion. Goats eat it; horses, cows, and sheep refuse it.—*Withering.*

19. Ranunculus. *R. acris. R. pratensis.*

*Ang.* Butter cups. Butter flower. Upright meadow crowfoot.

*Gen. Desc. As above.*

*Spec. Desc.* Cal. expanding, hairy, coloured. *Fruit-st.* cylindrical. *Leaves* hairy, three div. and many-cleft; segments black at the points. *Stem-l.* sitting, sheathing the joints. *Stam.* with hairs laid to. *Bloss.* yellow. *Meadows and pastures.* Bl. June, July.

*Use.* The whole plant is hot and caustic, readily and safely raising a blister, without causing stranguary or the like.—*Lightfoot.* It is very acrid, and blisters the skin easily.—*Withering.* The acrimony of this and many species of R. is so great, that on being applied to the skin they excite itching, redness, and inflammation, and even produce blisters, tumefaction and ulceration of the part; if chewed they corrode the tongue; and, taken into the stomach, bring on all the deleterious effects of an acrid poison. It is observed by Mr. Curtis, that even by pulling up this plant, and carrying it to some little distance, a considerable inflammation was excited in the palm of the hand in which it was held. This species is one of the most acrid, as well as the most common in England of the tribe. The acrimonious quality may be completely dissipated by heat; and when thoroughly dried, the plant becomes perfectly bland: and its virulency depends depends much on the situation in which it grows, and is greatly diminished in the cultivated plant. For medical purposes, this and others of the R. have chiefly been externally employed as *vesicatory*, and are said to have the advantage of a common blistering plaster, in producing a quicker effect, and never causing stranguary. On the other hand, it is said to be less certain in its operation, and sometimes occasions ulcers, which prove very difficult and troublesome to heal. Therefore their use seems to be applicable only to certain fixed pains, and such complaints as require a long continued topical stimulus, or discharge from the part in the way of an issue, which in various cases has been found to be a powerful remedy. For cases of its success in *chronic rheumatism*, and other complaints, See *Chesnau, Obs. Med. Bagliv. Op. p.* 113. *Stoerck Ann. Med. ii.* 125. The manner of using the plant, is to bruise it in a mortar, and to apply it to the skin as a poultice or plaster.—*Woodville.* Sheep and goats eat it; but horses, cows, and swine leave it untouched, though their pasture be ever so bare.—*Withering.*

20. Ranunculus. *R. bulbosus.*

*Ang.* Butter cups. Butter flower. Gold cup. Bulbous crowfoot. May flower

*Gen Desc. As above.*

*Spec. Desc. Root* bulbous, globular compressed, fibrous at the base. *Cal.* reflected. *Fruit st.* furrowed. *Stem* upright, many-flowered, 1 foot high, bare at base, at top leafy, branched, never throwing out succours. *Nectary* inversely heart-shaped, short, *(in the R. hirsutus it is oblong egg-shaped.) Petals,* bright yellow. *Meadows, pastures.* Bl. May.

*Use.* The whole plant is extremely acrid and corrosive, especially the fresh roots, which will readily raise a blister as safely as cantharides: notwithstanding this, the roots when boiled, become so mild as to be eatable.—*Lightfoot.* The bulb is formed above the bulb of the last year; when the flowers appear, the old bulb, in a dry soil, may be found in a state of decay, under the new one, and surrounded by fibres, but without the least appearance of suckers.—*Withering.*

21. Ranunculus. *R. arvensis.*

*Ang.* Corn crowfoot.

*Gen. Desc. As above.*

*Spec. Desc. Seeds* prickly. *Upper leaves* doubly compound, pound, strap-shaped. Whole plant, pale, upright, branched. *Flowers* small, pale yellow. *Cornfields.* Bl. June.

*Use.* It is so acrid, that three ounces of the juice killed a dog in four minutes; and that it poisons sheep which eat it; though, it is said, that in Italy, cows, horses, and sheep eat it greedily. Its growing solely in corn-fields, whence cattle are excluded, has, probably, prevented its doing mischief in this country.—*Withering.*

22. Helleborus, *H. fœtidus. Helleboraster.*

*Ang.* Bearsfoot. Setterwort. Oxheel. Stinking hellebore. Great black hellebore.

*Gen. Desc. Blos.* 0. Cal. five-leav. often coloured. *Nect.* two-lipped, tubular. Caps. like a legumen; many-seeded, rather upright, beaked.

*Spec. Desc. Stem* many-flowered, leafy. *Leaves* bird-footed, deep green. *Branches leaf-scales, floral-leaves,* and *flowers* pale greenish yellow. *Stipulæ* at the division of branches, oval-spear-shaped, embracing the stem, three-deep clefts tinged with purple. *Floral-leaf* oval-spear-shaped, entire, solitary, tinged with purple. *Flowers* numerous, globular, greenish, sometimes purplish. *Stem* 3 feet high. *Meadows, shady places, hedges.* Bl. Apr.

( To be continued. )

